# COVID-19 Pandemic: Another Source of Stress for Medical Students

**DOI:** 10.1192/j.eurpsy.2022.1260

**Published:** 2022-09-01

**Authors:** A.T. Pereira, C. Cabacos, A. Araújo, M.J. Soares, M.J. Brito, F. Carvalho, D. Mota, M. Bajouco, N. Madeira, M. Carneiro, A. Macedo

**Affiliations:** 1 Faculty of Medicine of University of Coimbra, Institute Of Psychological Medicine, Coimbra, Portugal; 2 Coimbra Institute for Biomedical Imaging and Translational Research, -, Coimbra, Portugal; 3 Centro Hospitalar e Universitário de Coimbra, Department Of Psychiatry, Coimbra, Portugal

**Keywords:** stress sources, Covid-19 pandemic, depression anxiety and stress scale, medical students

## Abstract

**Introduction:**

The COVID-19 pandemic has completely changed the experience of higher education with potentially negative consequences for students’ wellbeing.

**Objectives:**

To compare medicine/dentistry students’ depression/anxiety/stress levels before versus during the pandemic and to analyse the role of COVID-19-related stressors in their psychological distress.

**Methods:**

Students from the Faculty of Medicine University of Coimbra answered socio-demographic and personality questionnaires and the Depression, Anxiety and Stress Scale/DASS before (academic years 2016-2017-2018-2019 - SAMPLE1; n=1000) and during (September-December 2020 and January-March 2021 - SAMPLE2; n=650) the COVID-19 pandemic. Mean age (21.12±3.75), personality traits scores, and gender proportions (»75% girls) did not significantly differ between samples. SAMPLE2 also filled in the Fear of COVID-19 Scale and a new version of the Inventory of Sources of Stress During Medical Education/ISSDME, containing a COVID-19 -related dimension (restrictions on training and on socializing with friends/colleagues).

**Results:**

SAMPLE2 presented significantly higher mean scores of depression (3.89±3.55vs.3.33±3.34), anxiety (3.27±4.08vs.2.86±3.29), stress (7.07±5.72vs.6.18±4.59) and total DASS (12.28±10.55vs.13.65±11.13) than SAMPLE1 (all p<.05). Fear of COVID-19 was a significant predictor of DASS score (adjusted R2=2.9%, p<.001). COVID-19-related stressors continued explaining significant increments of DASS variance after controlling for each of the ISSDME dimensions: Course demands (R2 Change=1.8%), Human demands (2.5%), Lifestyle (2.3%), Academic competition (5.5%), and Academic adjustment (5.2%) (all p<.001).

**Conclusions:**

This study adds to the evidence of the negative impact of COVID-19 on students and emphasizes its pernicious role on medical students’ psychological distress, which is already higher due to the individual and academic stressors to which they are more exposed.

**Disclosure:**

No significant relationships.

